# Beyond vision: The emotional toll of advanced diabetic retinopathy

**DOI:** 10.1111/dme.70081

**Published:** 2025-05-26

**Authors:** Hellena Hailu Habte‐Asres, Clara Nartey, Sarah Afuwape

**Affiliations:** ^1^ Florence Nightingale Faculty of Nursing, Midwifery and Palliative Care, King's College London London UK; ^2^ Royal Free London NHS Foundation Trust London UK; ^3^ Moorfields Eye Hospital, NHS Foundation Trust London UK; ^4^ Department of Renal Medicine UCL London UK

Diabetes distress refers to the emotional and psychological burden of managing diabetes, including the pressures of daily self‐care and fear of complications. This burden may be heightened in individuals with advanced diabetic retinopathy (DR), where visual impairment, loss of independence and health‐related anxiety exacerbate distress. Although 30%–46% of people with diabetes experience distress,^1^, ^2^, ^3^ its prevalence in those with advanced DR, particularly those not undergoing surgery, remains underexamined.^4^ Given the increased risk of vision loss and its impact on quality of life,^3^, ^5^ this study aims to assess the prevalence of diabetes distress in this group and identify associated clinical and psychosocial factors.

This cross‐sectional study included individuals with diabetes referred to the nurse‐led diabetes service at Moorfields Eye Hospital between January 2023 and January 2024. Participants with ocular complications who consented and could complete the Diabetes Distress Scale (DDS‐17) were eligible, while those with cognitive impairments were excluded. We obtained approval from the hospital's Institutional Review Board (MEH‐1277) and the study adheres to the Declaration of Helsinki.

Diabetes distress was assessed using the 17‐item DDS‐17, which looks at four areas: emotional burden, regimen‐related distress, physician‐related distress and interpersonal distress.^6^ A mean score of 3 or more indicated severe distress. Sociodemographic and clinical information was gathered from electronic records.

Descriptive statistics summarised participant characteristics, χ^2^ tests compared distress across groups and logistic regression examined links with clinical and sociodemographic factors, adjusting for age and sex. Analyses were carried out using Stata version 17.

The study included 59 participants, with a mean age of 61.7 (±11.7) years. The sample was predominantly male (61%) and 52.5% were from a non‐white background. Nearly half (45.7%) of the participants lived in the most deprived areas. A majority had a raised body mass index (68.8%) and were hypertensive, with a mean systolic blood pressure of 137.4 mmHg. Most participants had varying levels of DR, with common grades including R1M1 and R3AM1 in the right eye and R2M1 and R3AM1 in the left. Seven participants had ocular complications, including cataracts, glaucoma and macular atrophy.

The mean total DDS score was 41.6 ± 20.1, with 32.2% of participants scoring ≥3, indicating diabetes distress. Emotional burden was the most common distress (35.5%), followed by physician‐related distress (37.3%) and regimen‐related distress (25.4%).

Unadjusted and adjusted analyses showed no significant association between diabetes distress and age, sex, BMI, blood pressure, HbA1c, ethnicity or deprivation (Table 1).

**TABLE 1:** Relationships between diabetes distress and sociodemographic/clinical variables.

Variable	Category	Unadjusted OR of diabetes distress (95% CI)^a^	Adjusted OR of diabetes distress (95% CI)^b^
Age	—	1 (0.9–1.0)	1 (0.9–1.0)
Sex	Female	1	1
	Male	0.4 (0.2–1.3)	0.4 (0.1–1.3)
Ethnicity	White	1	1
	Non‐White	0.9 (0.1–5.3)	0.8 (0.1–5.1)
	Unknown	3.0 (0. 5–18.6)	2.5 (0.4–16.7)
Index of multiple deprivation	1(Most deprived)	1	1
	2	0.4 (0.6–2.0)	0.3 (0.1–1.8)
	3	0.7 (0.1–4.3)	0.4 (0.1–3.1)
	4	0. 5 (0.1–3.0)	0.3 (0.1–2.7)
	5 (Least deprived)	0.3 (0.1–2.0)	0.2 (0.0–1.7)
Body Mass index	<25	1	1
	25 or <30	0.4 (0.1–1.6)	0.5 (0.1–2.0)
	≥30	1.4 (0. 4–5.1)	1.2 (0.3–3.4)
CKD status	No CKD	1	1
	CKD	1.0 (0.3–3.0)	1.1 (1.0–19.0)
SBP	—	1.0 (1.0–1.1)	1.0 (1.0–1.0)
DBP	—	1.0 (0.9–1.1)	1 (0.9–1.1)
HbA1c	—	1.0 (1.0–1.1)	1.0 (1.0–1.1)
Severe DR (R2, R3A 3RS)	No DR	1	1
	DR	1.2 (0. 4–4. 4)	1.7 (0.4–7.1)
Diabetic Maculopathy	M0	1	1
	M1	0.8 (0.3–2.8)	1.2 (0.3–4.7)

^a^ Model 1: crude association.

^b^ Model 2: adjusted for age, sex.

This study explored the link between diabetes distress and sociodemographic and clinical factors in individuals with diabetes and ocular complications. Around one‐third of participants (32.2%) reported diabetes distress, highlighting the need for integrated psychological support in this high‐risk group.^3^ This prevalence was lower than the 74.3% reported by Zhang et al.^4^ in patients undergoing DR surgery, likely due to differences in study populations and distress thresholds.

Despite many participants having advanced diabetic DR and maculopathy, no significant association was found with diabetes distress. This contrasts with Khoo et al.,^7^ who reported links between DR, visual impairment and poorer psychosocial outcomes such as anxiety, depression and lower mental health‐related quality of life. A bidirectional relationship was identified, but the underlying mechanisms remain unclear. These findings highlight the importance of early intervention to reduce both DR progression and diabetes distress.

Our results suggest that people with long‐standing diabetes and complications may adapt over time or benefit from support services that alleviate distress. Distress was not linked to HbA1c, indicating it is more influenced by treatment burden, healthcare experiences and psychosocial factors than glycaemic control. As others have noted, beliefs about illness and support networks may better predict well‐being than clinical measures.^3^ While a weak, non‐significant link between distress and CKD was observed, further research is needed to explore distress patterns in individuals with multiple complications. A pilot RCT by Rees et al.^8^ suggests problem‐solving therapy may improve both emotional and glycaemic outcomes.

## STRENGTHS AND LIMITATIONS

This study provides insights into diabetes distress in a high‐risk group with advanced complications. Strengths include the use of the validated DDS‐17 measure and both unadjusted and adjusted analyses. Limitations include a small sample size, cross‐sectional design and single‐centre recruitment.

## IMPLICATIONS AND FUTURE RESEARCH

These findings underscore the importance of integrating psychological support into diabetes care, particularly for those with advanced complications. Routine screening for distress could help identify individuals in need of additional support, enabling timely interventions such as structured education, peer support or psychological therapies.^9^

## CONCLUSION

Diabetes distress was common among individuals with ocular complications, affecting nearly one‐third of participants. No significant links were found with demographic or clinical factors, suggesting that distress may stem from broader psychosocial influences. Integrating psychological support into diabetes care may help address this burden. Further research is needed to understand its drivers and inform targeted support strategies.

## AUTHOR CONTRIBUTIONS

H.H.A. conceptualised the idea of undertaking the D.D. study within the department and prepared the manuscript. C.N. administered the questionnaire and assisted in collecting baseline data. S.A., as the senior author and guarantor of the work, oversaw the project, reviewed and revised the manuscript and provided expert knowledge in psychological health, particularly regarding distress.

## CONFLICT OF INTEREST STATEMENT

C.N. and S.A. have nothing to declare. HHA has received honoraria from AstraZeneca and Bayer.
